# *Emergomyces africanus* in Soil, South Africa

**DOI:** 10.3201/eid2402.171351

**Published:** 2018-02

**Authors:** Ilan S. Schwartz, Barbra Lerm, J. Claire Hoving, Chris Kenyon, William G. Horsnell, W. Joan Basson, Patricia Otieno-Odhiambo, Nelesh P. Govender, Robert Colebunders, Alfred Botha

**Affiliations:** University of Manitoba, Winnipeg, Manitoba, Canada (I.S. Schwartz); University of Antwerp, Antwerp, Belgium (I.S. Schwartz, R. Colebunders);; Stellenbosch University, Stellenbosch, South Africa (B. Lerm, W.J. Basson, A. Botha);; University of Cape Town, Cape Town, South Africa (J.C. Hoving, C. Kenyon, W.G. Horsnell, P. Otieno-Odhiambo, N.P. Govender);; Institute of Tropical Medicine, Antwerp (C. Kenyon);; University of Birmingham, Birmingham, UK (W.G. Horsnell);; CNRS-University of Orleans and Le Studium Institute for Advanced Studies, Orléans, France (W.G. Horsnell);; National Institute for Communicable Diseases, Johannesburg, South Africa (N.P. Govender)

**Keywords:** mycoses, environmental microbiology, emmonsia, environmental microbiology, mycoses, mice, fungal infections, soil, Emergomyces africanus, South Africa, fungi

## Abstract

We detected *Emergomyces africanus*, a thermally dimorphic fungus that causes an HIV-associated systemic mycosis, by PCR in 18 (30%) of 60 soil samples from a wide range of habitats in South Africa. Direct and indirect culture techniques were unsuccessful. Experimental intraperitoneal inoculation of conidia induced murine disease.

The newly described thermally dimorphic fungal genus *Emergomyces* comprises human pathogens that cause systemic mycoses in immunocompromised persons globally (*1*). Among these fungi, *Emergomyces africanus* (formerly *Emmonsia* sp. [*2*]) is the species responsible for the most human disease. HIV-associated emergomycosis is the most common endemic mycosis in South Africa and is associated with a high case-fatality ratio (*3*,*4*).

Although an environmental reservoir for *Es*. *africanus* has not been established, soil is presumed to harbor the mycelial phase (*2*). We tested soils in South Africa for *Es. africanus* by using molecular- and culture-based methods.

## The Study

We collected 60 soil samples from various soil habitats around South Africa by convenience sampling; 82% percent of samples came from the Western Cape Province, with the remaining samples from Gauteng (7%), Eastern Cape (7%), KwaZulu-Natal (2%), and Northern Cape (2%) provinces. For each sample, we used sterile, plastic tubes to collect ≈100 mL of topsoil.

We extracted DNA from soil by using the ZR Soil Microbe DNA Miniprep Kit (Zymo Research, Irvine, CA, USA). DNA extraction was successful for 56 soil samples (93%). We subjected extracted genomic DNA (gDNA) to a nested PCR. To amplify the internal transcribed spacer (ITS) region of the ribosomal RNA, we used the universal primers ITS1 and ITS4 in the first reaction (*5*). We used an Applied Biosystems 2720 Thermal Cycler (Foster City, CA, USA); thermocycling conditions consisted of 95°C for 5 min, 30 cycles of 95°C for 30 s, 52°C for 30 s, 72°C for 45 s, and 72°C for 7 min. We subjected PCR products to amplification by using *Es. africanus*–specific primers (forward, 5′-CCTGGTTTGGGGAGAGGGGT-3′; reverse, 5′-CCGGGGGAGCTCTTGGCTCT-3′), followed by electrophoresis on a 2% agarose gel. We performed amplification as described, except with an annealing temperature of 57°C. PCR mixtures consisted of 10 µL 2× KAPA Taq ReadyMix (KAPA Biosystems, Wilmington, MA, USA); 1 µL of each primer (10 µmol/L; Inqaba Biotechnical Industries, Pretoria, South Africa); and 1 µL of extracted gDNA or ITS PCR product, in a final reaction volume of 20 µL. We sequenced amplified products and compared them using BLAST (https://blast.ncbi.nlm.nih.gov/Blast.cgi). The PCR could detect as few as 10^2^–10^4^ conidia/10 g of soil (Technical Appendix).

**Figure 1:**
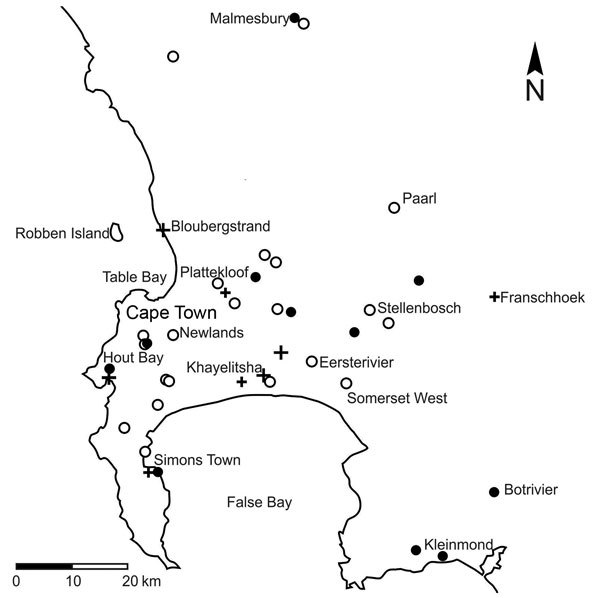
Results of molecular tests for the presence of *Emergomyces africanus* in soil samples in relation to residential locations of 14 patients diagnosed with emergomycosis (*6*), Cape Peninsula, Western Cape Province, South Africa. Black circles indicate *Es. africanus* detected in soil sample; white circles indicate *Es. africanus* not detected in soil sample; plus signs indicate residential locations of patients with emergomycosis. A larger cross indicates >1 infected patient at that particular location.

We plotted results of molecular testing and residential postal codes of persons with confirmed infections (Figure 1). We detected *Es. africanus* DNA in 18 (32%) of 56 soil samples representing all types of soil habitats tested (Table).

**Table:** Results of molecular-based detection of *Emergomyces africanus* in 60 soil samples, by province and type of soil habitat, South Africa*

Soil habitat	Western Cape	Eastern Cape	Gauteng	KwaZulu-Natal	Northern Cape	Total
Garden	6/30	0/2	1/4	–	–	7/36
Agricultural	3/5	–	–	–	–	3/5
Compost	3/5	–	–	–	–	3/5
Disturbed	1/2	0/2	–	0/1	–	2/5
Fynbos	1/2	–	–	–	–	1/2
Veld	1/1	0/1	–	–	0/1	1/3
Rotting tree	1/1	–	–	–	–	1/1
Unknown	1/3	–	–	–	–	1/3
Total	17/49	0/4	1/4	0/1	0/1	18/60

*Data represent number of samples in which *Es. africanus* was detected by nested PCR/total number of samples. Soil habitats: garden, soil from private gardens; agricultural, soil used for farming purposes; compost, soil rich in compost; disturbed, nutrient-poor uncultivated soil subjected to anthropogenic activities; fynbos, soil from a natural indigenous vegetation type endemic to the Cape Floristic region; veld, soil from grassland or uncultivated land; rotting tree, decaying woody debris; unknown, soil from unknown origin. –, sample not taken.

We used soil dilution plates prepared with Sabouraud agar (40 g/L glucose [Merck, Darmstadt, Germany], 10 g/L peptone [Merck], and 15 g/L agar) supplemented with 0.2 g/L chloramphenicol (Sigma-Aldrich Chemie GmbH, Steinheim, Germany) to culture *Es. africanus* from 4 randomly selected soil samples. We incubated the resulting spread plates at 26°C, inspecting plates daily for 1 week and then twice weekly for an additional 3 weeks. All culture plates were rapidly overgrown by filamentous fungi other than *Es. africanus.*

To overcome rapid contamination, we used indirect culture methods. First, we used the flotation method adapted from Larsh et al. (*7*) to separate the conidia from other particles in the soil (Technical Appendix). We plated the resulting soil suspensions on Sabouraud agar and brain heart infusion (BHI) plates and incubated them at 26°C, conducting daily examinations for fungal colonies resembling *Es. africanus* (*1*). This preparation also resulted in rapid contamination of all plates.

**Figure 2:**
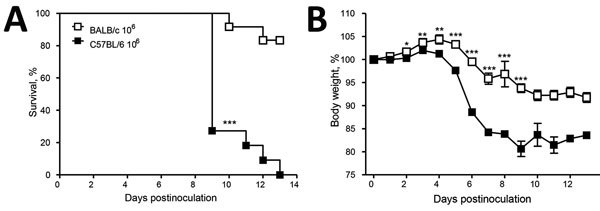
Infection of mice with *Emergomyces africanus.* In a proof-of-principle study, C57BL/6 and BALBc mice were inoculated intraperitoneally with 10^6^ conidia (*Es. africanu*s CAB 2141, a clinical isolate) in saline. Mice were weighed and monitored twice daily for distress. Both mouse strains had symptom onset, with C57BL/6 mice showing significantly more severe disease pathophysiology in response to the high dose of *Es. africanus* (demonstrated by reduced survival and increased weight loss). Data represent 2 pooled experiments (n = 8 [panel A] and n = 2 [panel B] combined), mean +SD of the mean. p values were determined by using unpaired 2-tailed Student *t*-test or 1-way analysis of variance using a Bonferroni posttest (GraphPad Prism version 5). Values of p<0.05 were considered significant. *p<0.05; **p<0.01; ***p<0.001 (C57BL/6 compared with BALB/c mice).

Thereafter, we passaged soil suspensions through mice to screen out nonpathogenic soil organisms (*8*; Technical Appendix). Animal studies were approved by the University of Cape Town’s Animal Ethics Committee (protocol 016–002). We created soil suspensions by using the flotation method and sampling from the bottom third of the column; penicillin G (1,000 IU/mL) and gentamicin (0.1 mg/mL) were included in the solution. We inoculated 1 mL of soil suspension intraperitoneally into each of 4 BALB/c or C57BL6 mice. We euthanized the mice after 2 weeks and plated livers, spleens, or both onto Sabouraud agar plates with and without chloramphenicol, which we then incubated at 30°C and 35°C –37°C. We inspected plates as described previously. Pilot studies demonstrated that this method could detect as few as 10^2^ conidia in 10 g of soil (Technical Appendix). Notably, in a pilot study in which BALB/c and C57BL/6 mice were challenged with graded doses *of Es. africanus* conidia, genetic background of mice influenced host susceptibility to the organism; C57BL/6 mice were more sensitive to infection and had significantly higher mortality and weight loss in response to the high dose of 10^6^ conidia compared with BALB/c mice (Figure 2).

We screened 26 soil samples for the presence of *Es. africanus* by using mouse passage. These samples included all 18 soil samples in which *Es. africanus* was detected by nested PCR, as well 8 soil samples that were PCR-negative. None of these samples, however, led to the isolation of *Es. africanus* through mouse passage. 

## Conclusions

*Es. africanus* is a newly described dimorphic fungal pathogen and causes an important HIV-associated systemic mycosis in South Africa (*9*). Many aspects of this organism remain unknown, including its ecologic niche. Our findings demonstrate that *Es. africanus* is present in a high proportion of soil samples collected from a range of habitats in South Africa, suggesting that soil might be a natural reservoir for this pathogen.

The isolation of pathogenic fungi from soil is challenging. Soil naturally contains a vast array of bacteria, viruses, fungi, and protozoa, all of which can interfere with or contaminate culturing the organism of interest (*8*). Since 1932, when Stewart and Meyer first cultured *Coccidioides immitis* from soil (*10*), flotation and animal passage has been the most robust method to isolate pathogenic fungi from soil. However, animal passage is laborious and expensive, can take months of turnaround time, requires special animal facilities, and results in discomfort and loss of life to laboratory animals, necessitating stringent ethics review (*11*).

Molecular detection is a valuable tool for establishing the presence of genetic material in environmental samples (*11*). In addition to high sensitivity, molecular detection has the advantages of being easy to apply, inexpensive, and rapid, and it can be performed in most laboratories. Alternatively, molecular detection lacks specificity because it cannot determine the viability (and hence infectivity) of the detected target (*11*). In our study, mouse passage of soil samples shown by nested PCR to contain *Es. africanus* genetic material did not result in the isolation of this fungus.

We have demonstrated that experimental infection with *Es. africanus* can produce pathology in mice. Moreover, susceptibility to disease appears to be mouse strain–dependent, with C57BL/6 mice being more susceptible than BALB/c mice.

This study has some limitations. The number of samples, and especially those from outside Western Cape Province, was relatively small, limiting inferences about the geographic range of *Es. africanus* in the environment. Moreover, our method of convenience sampling is prone to sampling bias. Nonetheless, this study is instructive for future ecologic studies, which should use random sampling to refine knowledge of the ecologic niche of this fungus.

In conclusion, this study demonstrates that *Es. africanus* can be frequently detected in a wide range of soils in South Africa. Moreover, our findings support the hypothesis that soil serves as a reservoir for this pathogen.

Technical Appendix Methodology and validation of molecular and culture-based methods for detection of *Emergomyces africanus* from soil, South Africa.
