# Supplementary material for: Emergomyces africanus in Soil, South Africa
Source: Emerg Infect Dis. 2018 Feb;24(2):377–80. doi: 10.3201/eid2402.171351 (PMC5782882; doi:10.3201/eid2402.171351)
Supplement: Technical Appendix — Methodology and validation of molecular and culture-based methods for detection of Emergomyces africanus from soil, South Africa. [file 17-1351-Techapp-s1.pdf]

Article DOI: https://doi.org/10.3201/eid2402.171351

Emergomyces africanus in Soil, South Africa

Technical Appendix

Establishing Sensitivity of Molecular Detection Method

We used artificial inoculation of soil to determine the limits of the PCR. First, to remove microbial biomass including possible *Emergomyces africanus* cells, 1 kg of sandy soil was acid-washed by submerging in 0.1 M HCl (Merck, Darmstadt, Germany) for 14 h, whereafter the soil was rinsed 3 times with distilled water and oven dried at 80°C. To confirm the absence of *Es. africanus* gDNA in the acid-washed soil, DNA extractions were conducted on triplicate soil sub-samples (0.25 g). Extracted gDNA was subjected to nested PCR and tested for presence of a PCR product by visualization using agar gel electrophoresis. The lack of nested PCR product indicated the absence of *Es. africanus* DNA in the acid-washed soil.

A suspension of *Es. africanus* conidia was subsequently prepared using a 28-day-old fungal culture of *Es. africanus* CAB 2141 (a clinical isolate) grown on brain heart infusion (BHI; Merck) agar at 26°C. In brief, 5 mL saline Tween 80 solution (0.89% sodium chloride and 0.01% Tween 80) was added to the BHI agar plate (diameter, 90 mm), whereafter an inoculation loop was used to gently scrape the surface of the culture. The resulting conidial suspension in the Tween 80 and saline solution was aseptically filtered through a loose plug of glass wool (*1*) in a Pasteur pipette into a sterile test tube, whereafter the conidia in the filtrate were counted using a hemocytometer (Improved Neubauer, Marienfeld Superior, Germany). Samples of acid-washed soil (10 g) were then added to conical flasks (50 mL) and artificially inoculated with 10^2, 10^3, 10^4 and 10^6 conidia, respectively. A negative control consisted of 10 g of acid-washed soil without added conidia. All flasks were incubated at 26°C for 24 h with regular shaking to allow time for conidial binding to soil particles (*2*). After incubation, DNA extractions were conducted in duplicate on each of the artificially inoculated soil samples. Thereafter, extracted DNA was

subjected to nested PCR. Amplified products were sequenced and compared to the available sequences on the GenBank database.

A nested PCR product of the expected size (≈400 bp) was obtained from extracted DNA originating from 10 g of soil artificially inoculated with 10^2 conidia. The product was visualized on a 2% agarose gel but its concentration was too low for sequencing. Nested PCR products, as visualized by agarose gel electrophoresis, were also obtained from DNA originating from 10 g of soil inoculated with 10^4 and 10^6 conidia, respectively (Technical Appendix Figure). Sequencing and BLAST analysis of the nested PCR product, representative of the soil inoculated with 10^4 conidia, confirmed the presence of *Es. africanus*. From these results, it seems that the detection limits of the method used in the experimentation was between 10^2 and 10^4 conidia per 10 g of soil.

Flotation Method

In an attempt to separate *Es. africanus* conidia from other particles in the soil, we used the flotation method adapted from Larsh *et al* (*3*). The method entails preparing 100 mL soil suspension in a saline Tween 80 solution, from 10 g of soil within a 100-mL measuring cylinder. First, a pilot experiment was conducted to localize where in the column the conidia settled 1 hour after mixing with the saline Tween 80. For this, we spiked sterilized soils with known quantities of conidia produced by *Es. africanus* CAB 2141. Conidia of a 21-day old plate culture, on Sabourad (SAB) agar incubated at 26°C, were harvested using the filtered saline Tween 80 method described above. After using a hemocytometer to count conidia suspended in filtered saline Tween 80, 10^6 conidia were inoculated into 10 g of sterilized soil contained in a measuring cylinder. Also, dilution plates were prepared with SAB agar to determine the numbers of culturable conidia in the conidial suspension. These viability plates were incubated at 26°C; developing fungal colonies were counted twice weekly for 4 weeks.

Two soils were used in the experimentation: sandy soil collected from and representative of that which predominates in the Cape Flats, an area of the Western Cape where clinical cases of *Es. africanus* infection have been diagnosed (*4*); and compost soil from the Winelands area, Western Cape. Both soils were sterilized by autoclaving twice at 121°C for 20 min. In each case,

the autoclaved soil was cooled to ca. 25°C, whereafter it was transferred to a measuring cylinder and inoculated as described above.

Each measuring cylinder, containing the 10 g of inoculated soil, was aseptically filled to a total volume of 100 mL with saline Tween 80. The cylinder was subsequently covered with sterilized aluminum foil and agitated with a Vortex Genie 2 (Scientific Industries, Bohemia, NY, USA) for 3–5 minutes. The resulting soil suspension was allowed to settle for 1 hour, whereafter 5 mL sub-samples of the suspension was collected from the top, middle, and bottom of the column. For the compost soil, these aliquots were each aseptically filtered through a loose plug of glass wool in a Pasteur pipette; this step was omitted for the sandy soil because few grossly visible soil particles remained suspended. The remaining soil suspension in each measuring cylinder was subdivided in 2 and concentrated via centrifugation (2500G; 30 min), whereafter 10 mL combined concentrated suspension (5 mL from the bottom of each centrifuge tube) was retained; the remainder was discarded. Finally, ≈1 mL of soil was removed from the bottom of the cylinder, suspended in 9 mL saline Tween 80, and agitated. This suspension was passed through glass-wool for the compost soil, but the filtering step was omitted for the sandy soil, as above. The culturable conidia, suspended in each of the above-mentioned 5 sub-samples, were enumerated using plate counts. From each sub-sample, 0.1 mL was plated onto each of 10 BHI plates and incubated at 26°C. Colonies were counted after 8 days. The rate of recovery of conidia was calculated as total colonies from all sample locations divided by the expected number based on colony counts on the viability plates (Technical Appendix Table 1).

Flotation of Sterilized Soil Seeded with *Es. africanus* Conidia

Flotation resulted in a low recovery of *Es. africanus* conidia (Technical Appendix Table 1). The total proportions of inoculated conidia recovered from the sterilized sandy and compost soil samples were 4.7% and 10.0%, respectively. The total proportions of conidia that were floated away from the sedimented soil were 96.1% and 85.9% for the sandy and compost soil, respectively.

For selected soil samples that were positive by molecular detection, we performed the flotation method, as described above but only sampling from the bottom third of the column based on the results of the pilot experiment. The sample was then plated on SAB and BHI plates

and incubated at 26°C, with daily examination for developing fungal colonies resembling those of *Es. africanus* (*1*).

Mouse Inoculation

Animal studies were approved by the University of Cape Town's Animal Ethics Committee (protocol 016–002).

To overcome rapid overgrowth of soil dilution plates by filamentous fungi other than *Es. africanus*, we passaged filtered soil suspensions through mice obtained from the University of Cape Town Research Animal Facility (*5*). First, we tested whether BALB/c and C57BL/6 mice could be infected with *Es. africanus* by inoculating them intraperitoneally (i.p.) with varying doses of conidia (from 10^2-10^6 in 1 mL saline). Mice were euthanized after 2- and 4-weeks and livers and spleens were plated onto SAB incubated at 30°C and 35–37°C, respectively. Plates were inspected daily for developing fungal colonies.

Conidia suspended in saline and inoculated into wild-type mice could be detected as developing colonies upon culture of their spleens and livers. Here, the genetic background of the mice susceptibility to the organism: C57BL/6 mice were more sensitive to infection with significantly higher mortality and weight loss in response to the high dose of 10^6 conidia compared to BALB/c mice (Figure 2 in main manuscript). The organisms were visualized in the spleen of C57BL/6 mice that were euthanized after reaching 20% weight loss (data not shown). C57BL/6 mice inoculated with 10^2-10^5 conidia in saline handled the infection well (i.e., they did not lose weight and did not die or require euthanasia) but *Es. africanus* could still be cultured from spleen and liver after i.p. inoculation with as few as 10^2 conidia.

Inoculation of Mice with Soil Suspensions Derived from Sterilized and Unsterilized Soils Seeded with *Es. africanus* Conidia

Next, we tested whether the flotation method plus mouse passage could recover *Es. africanus* from unsterilized soil. In each case, 10 g of soil aliquots were artificially inoculated with 10^2, 10^3, 10^4 and 10^6 *Es. africanus* conidia, respectively. A soil suspension of each aliquot was subsequently prepared in a 100-mL measuring cylinder as described above, but with the addition of penicillin G (1000 IU/mL) and gentamicin (0.1 mg/mL) to the saline Tween 80. After

an hour, *Es. africanus* conidia were obtained from the suspension, by extracting a 5-mL sample from the bottom third of the column (based on the pilot data from the flotation experiment), whereafter the 5-mL conidial suspension was filtered through a loose plug of glass wool in a Pasteur pipette. We then inoculated 1 mL of the filtered suspension i.p. into each of 4 C57BL/6 mice and 4 BALB/c mice. The mice were pre-treated with subcutaneous carprofen (5 mg/kg), a non-steroidal anti-inflammatory analgesic, based on a previous report that suggested that this may have mitigated adverse reactions in mice exposed to Arizona soil (*6*). Mice were euthanized after 2- and 4-weeks and livers and spleens were plated onto SAB (with and without chloramphenicol) and BHI plates, incubated at 30°C and 35–37°C, respectively. Plates were inspected daily for developing fungal colonies resembling *Es. africanus*. The experiment was terminated after 4 weeks and the identities of colonies resembling *Es. africanus* were confirmed by sequence analyses of ITS region.

The limit of detection for mouse inoculation of unsterilized soil suspensions appeared to be 10^2 and 10^4 conidia per 10 g of soil for BALB/c and C57BL/6 mice, respectively (Technical Appendix Table 2). The procedure was tolerated by the mice, and no animals died or required euthanasia before the planned chronological endpoint.

Using Mouse Passage to Screen Soil Samples

A subset of 26 soil samples, from a set of 60 samples collected from around South Africa were subsequently screened for the presence of culturable *Es. africanus* via the above-mentioned mouse passage. Each soil sample was prepared using the flotation method as described above, inoculated i.p. into each of 4 C57BL/6 mice, 4 BALB/c mice, or both. Mice were pretreated with carprofen, as above, and euthanized after 2 weeks whereafter spleens and/or livers were plated onto SAB agar, with and without chloramphenicol, and incubated at 30°C and 35–37°C. Plates were monitored and fungal growth identified as above.

Monitoring and Euthanasia of Mice

After inoculation, mice were monitored at least daily for signs of illness or need for euthanasia. We defined the humane endpoint for animals as loss of 20% bodyweight; loss of 10% bodyweight sustained over 3 days plus another sign of clinical stress, such as hunching,

tachypnea, anorexia; or, clinical signs of distress such as lethargy and absence of response to being approached or touched by the monitor. Upon reaching the humane or chronological endpoints, mice were anesthetized with inhalation of halothane (5% in air), and euthanized by inhalation of CO_2 (10%–20% fill rate per minute) followed by cervical dislocation.

Epidemiologic Data

Residential postal codes of incident cases of disease caused by *Es. africanus* diagnosed in the Western Cape between Dec 2014 and Feb 2016 were collected. The locations representing these postal codes were plotted in comparison to study sample sites to ensure appropriate sampling in areas where persons are affected by *Es. africanus* infection. Patient enrolment and data collection was approved by human research ethics committees of University of Cape Town (HREC 138/2014), Stellenbosch University (N14/02/011), Institute of Tropical Medicine and University of Antwerp (ITG 926/14), and the institutional review boards of provincial hospital involved.

References

1. Yarrow D. Methods for the isolation, maintenance and identification of yeasts. In: Kurtzman CP, Fell JW, eds. The yeasts, a taxonomic study. 4th edition. Amsterdam: Elsevier Science B.V.; 1998. p. 77–100.

2. Huang J, Kang Z. Detection of *Thielaviopsis basicola* in soil with real-time quantitative PCR assays. Microbiol Res. 2010;165:411–7. PubMed http://dx.doi.org/10.1016/j.micres.2009.09.001

3. Larsh HW, Hinton A, Furcolow ML. Laboratory studies of *Histoplasma capsulatum*. III. Efficiency of the flotation method in isolation of *Histoplasma capsulatum* from soil. J Lab Clin Med. 1953;41:478–85. PubMed

4. Schwartz IS, Govender NP, Corcoran C, Dlamini S, Prozesky H, Burton R, et al. Clinical characteristics, diagnosis, management and outcomes of disseminated emmonsiosis: a retrospective case series. Clin Infect Dis. 2015;61:1004–12. PubMed http://dx.doi.org/10.1093/cid/civ439

5. Emmons CW. The isolation from soil of fungi which cause disease in man. Trans N Y Acad Sci. 1951;14:51–4. **PMID: 14901429**

6. Barker BM, Tabor JA, Shubitz LF, Perrill R, Orbach MJ. Detection and phylogenetic analysis of *Coccidioides posadasii* in Arizona soil samples. Fungal Ecol. 2012;5:163–76. http://dx.doi.org/10.1016/j.funeco.2011.07.010

Technical Appendix Table 1. Efficiency of the flotation method in recovery of culturable *Es. africanus* CAB 2141 from sterilized soil artificially inoculated with conidia

	No. of recovered colonies (% of total recovered)	
Sample location from column	Sandy soil	Compost soil
Top of column	239 (10.3)	373 (15.8)
Middle of column	655 (28.2)	673 (28.4)
Bottom of column	597 (25.7)	896 (37.9)
Centrifuged soil suspension	743 (32.0)	92 (3.9)
Sedimented soil	91 (3.9)	333 (14.1)
Total	2325 (100)	2367 (100)

*Number of culturable conidia in inoculum for sandy and compost soil samples were 4.96 $x10^4$ and 2.37 $x10^4$, respectively

Technical Appendix Table 2. Recovery of *Es. africanus* CAB 2141 from mouse passage with unsterilized soil artificially inoculated with conidia*

	30°C								37°C			
Group, x 4 mice	1	1 (drugs)	2	2 (drugs)	3	3 (drugs)	4	4 (drugs)	1	2	3	4
BALB/c												
Control	X	X	X	X	BC + FC	C	FC	X	X	FC	FC	FC
10^2	X	X	BC	X	C	BC	EA	EA	BC	X	BC	X
10^3	EA	EA	EA	EA	EA	EA	EA	EA	EA	FC	BC	EA
10^4	EA	EA	EA	EA	EA	EA	EA	EA	EA	EA	EA	EA
10^6	EA	EA	EA	EA	EA	EA	EA	EA	EA	BC	EA	EA
C57BL/6												
Control	FC	BC	BC	FC	X	FC	BC	BC	BC	BC	X	X
10^2	X	BC	FC	FC	X	X	X	BC	BC	X	BC	X
10^3	FC	X	BC	X	X	X	C	X	FC	FC	FC	FC
10^4	EA	EA	EA	EA	EA	EA	EA	EA	FC	EA	EA	EA
10^6	BC	BC	EA	EA	EA	EA	EA	EA	X	EA	EA	EA

*Livers and spleens of each mouse were plated onto 3 different isolation media that were subsequently incubated at different temperatures: Sabouraud's agar with dextrose with and without chloramphenicol (0.05 g/L; designated here as "drugs") incubated at 30°C, and Sabouraud's agar with dextrose incubated at 37°C. EA, *Es. africanus* isolated; X, no growth; BC, bacterial contamination, FC: fungal contamination, C: contamination not specified.

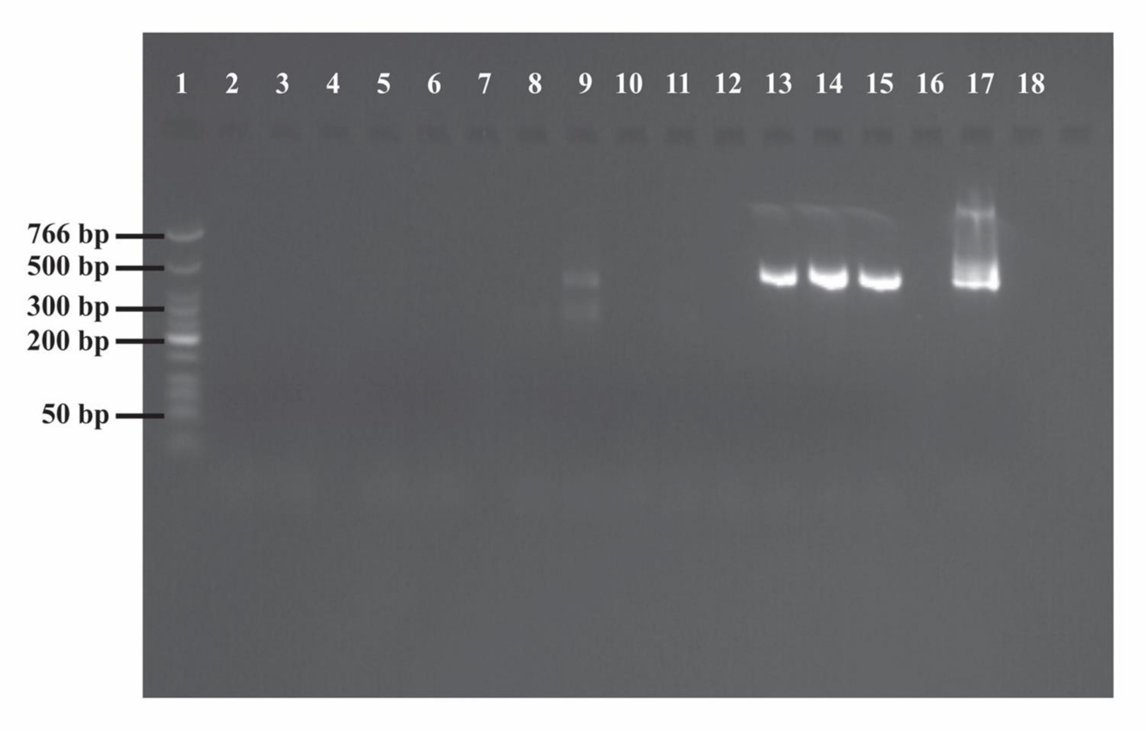

Technical Appendix Figure. UV image of nested PCR products originating from gDNA of acid-washed soil subsamples (10 g) artificially inoculated with 10^2, 10^3, 10^4 and 10^6 *Es. africanus* conidia, respectively. Products were separated with electrophoresis on a 2% agarose gel. Lane 1, Quick- Load Low Molecular Weight DNA Ladder (New England Biolabs, Massachusetts). Lanes 2 and 3, PCR negative controls containing no DNA. Lane 4, blank. Lanes 5 and 6, artificial soil inoculation negative controls (without added conidia). Lane 7, blank. Lanes 8 and 9, acid-washed soil inoculated with 10^2 conidia. Lanes 10 and 11, acid-washed soil inoculated with 10^3 conidia. Lanes 12 and 13, acid-washed soil inoculated with 10^4 conidia. Lanes 14 and 15, acid-washed soil inoculated with 10^6 conidia. Lane 16, blank. Lane 17, PCR positive control containing gDNA of *Es. africanus* CAB 2141. Lane 18, blank.
